# Supplementary material for: The causal relationship between the human gut microbiota and pyogenic arthritis: a Mendelian randomization study
Source: Front Cell Infect Microbiol. 2024 Nov 26;14:1452480. doi: 10.3389/fcimb.2024.1452480 (PMC11629706; doi:10.3389/fcimb.2024.1452480)
Supplement: Supplementary file 1 [file DataSheet1.zip › mendelian test/ebi-a-GCST90027722.csv_leave_one_out.pdf]

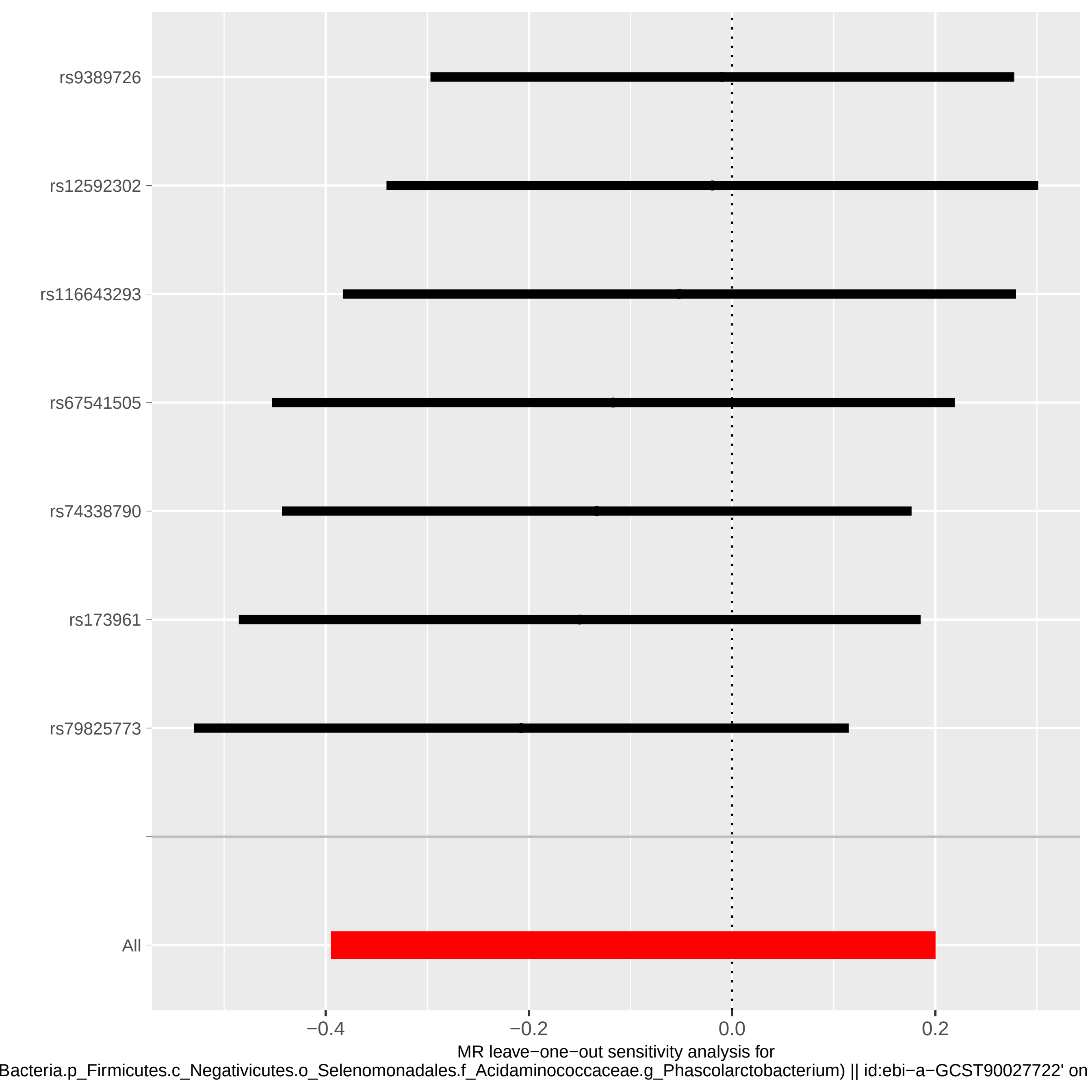
rs9389726
rs12592302
rs116643293
rs67541505
rs74338790
rs173961
rs79825773
All
−0.4
−0.2
0.0
0.2
MR leave−one−out sensitivity analysis for
Bacteria.p_Firmicutes.c_Negativicutes.o_Selenomonadales.f_Acidaminococcaceae.g_Phascolarctobacterium) || id:ebi−a−GCST90027722' on
